# Discovery of retinoic acid receptor agonists as proliferators of cardiac progenitor cells through a phenotypic screening approach

**DOI:** 10.1002/sctm.19-0069

**Published:** 2019-09-11

**Authors:** Lauren Drowley, Jane McPheat, Anneli Nordqvist, Samantha Peel, Ulla Karlsson, Sofia Martinsson, Erik Müllers, Anita Dellsén, Sinead Knight, Ian Barrett, José Sánchez, Björn Magnusson, Boris Greber, Qing‐Dong Wang, Alleyn T. Plowright

**Affiliations:** ^1^ Research and Early Development, Cardiovascular, Renal and Metabolism BioPharmaceuticals R&D Gothenburg Sweden; ^2^ Discovery Sciences R&D, AstraZeneca Gothenburg Sweden; ^3^ Discovery Sciences R&D, AstraZeneca Cambridge UK; ^4^ Human Stem Cell Pluripotency Laboratory Max Planck Institute for Molecular Biomedicine Münster Germany

**Keywords:** cell proliferation, gene expression, high throughput screening assays, induced pluripotent stem cells, nuclear receptors, small molecule libraries

## Abstract

Identification of small molecules with the potential to selectively proliferate cardiac progenitor cells (CPCs) will aid our understanding of the signaling pathways and mechanisms involved and could ultimately provide tools for regenerative therapies for the treatment of post‐MI cardiac dysfunction. We have used an in vitro human induced pluripotent stem cell‐derived CPC model to screen a 10,000‐compound library containing molecules representing different target classes and compounds reported to modulate the phenotype of stem or primary cells. The primary readout of this phenotypic screen was proliferation as measured by nuclear count. We identified retinoic acid receptor (RAR) agonists as potent proliferators of CPCs. The CPCs retained their progenitor phenotype following proliferation and the identified RAR agonists did not proliferate human cardiac fibroblasts, the major cell type in the heart. In addition, the RAR agonists were able to proliferate an independent source of CPCs, HuES6. The RAR agonists had a time‐of‐differentiation‐dependent effect on the HuES6‐derived CPCs. At 4 days of differentiation, treatment with retinoic acid induced differentiation of the CPCs to atrial cells. However, after 5 days of differentiation treatment with RAR agonists led to an inhibition of terminal differentiation to cardiomyocytes and enhanced the proliferation of the cells. RAR agonists, at least transiently, enhance the proliferation of human CPCs, at the expense of terminal cardiac differentiation. How this mechanism translates in vivo to activate endogenous CPCs and whether enhancing proliferation of these rare progenitor cells is sufficient to enhance cardiac repair remains to be investigated.

## Graphical abstract

An image‐based phenotypic screen, using a readout of nuclear count to measure proliferation, identified retinoic acid receptor agonists as potent proliferators of induced pluripotent stem cell‐derived cardiac progenitor cells. The cardiac progenitor cells retained their progenitor phenotype following proliferation and the retinoic acid receptor agonists did not proliferate human cardiac fibroblasts, the major cell type in the heart.

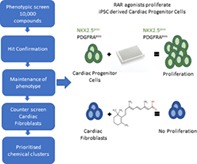

1

**Significance statement**

Progenitor cells are rare and difficult to isolate, and little is known about the molecular mechanisms required for their proliferation and differentiation. Identification of small molecules that selectively proliferate cardiac progenitor cells will aid in the understanding of the signaling mechanisms involved and could provide tools for regenerative therapies for the treatment of cardiac dysfunction. A phenotypic screen identified retinoic acid receptor agonists as potent proliferators of induced pluripotent stem cell‐derived cardiac progenitor cells. The agonists did not proliferate human cardiac fibroblasts, the major cell type in the heart but did proliferate human pluripotent stem cells, HuES6, in a time‐of‐differentiation‐dependent manner.

## 2 INTRODUCTION

Heart failure is a growing problem with a morbidity and mortality rate similar to cancer. It has been demonstrated that the heart has a low but detectable level of regenerative capacity in the adult setting,^1^ and efforts are ongoing to harness this power to truly reverse disease. A number of putative progenitor populations have been identified in the adult heart by independent groups^2^, ^3^, ^4^, ^5^, ^6^, ^7^, ^8^ and although these cells have limited cardiomyogenic potential,^9^, ^10^, ^11^ they may contribute to cardiac repair via directly forming endothelial cells and vasculature as well as via paracrine mechanisms.^9^, ^10^, ^11^, ^12^, ^13^ Due to their rarity and the difficulty of isolation, little is known about the molecular mechanisms required for their proliferation and differentiation.

We have previously published on a population of cardiac progenitor cells (CPCs) generated from human pluripotent stem cells during the differentiation process to cardiomyocytes and their use as tools in drug discovery.^14^ These cells express the cellular markers KDR (vascular endothelial growth factor [VEGF] receptor 2), PDGFR‐α (platelet‐derived growth factor receptor alpha), and NKX2.5. To date, only a handful of chemical mediators of cardiogenesis have been reported.^15^, ^16^ These compounds are useful tools for generating cardiomyocytes and blood vessels and for understanding the underlying biology and timing of the signaling pathway intervention. Here, we illustrate using our previously established screening method that we have identified a novel role of retinoic acid (RA) signaling in the proliferation of CPCs.

RA plays many critical roles during development, including acting as a morphogen, inducing commitment of precursor cells and thereby mediating tissue patterning.^17^, ^18^ The well‐established programs where RA is involved include both proliferation and differentiation. Inside the cells, RA is transported to the nucleus, where it binds to retinoic acid receptor (RAR) and retinoic X receptor (RXR) heterodimers, which in turn bind to sequence specific DNA elements known as RA response elements (RAREs). The receptors undergo conformational changes leading to corepressor displacement and recruitment of coactivators promoting active transcription of RAR target genes.^19^ In untreated stem cells, RAR primary target genes such as Hoxa1, Cyp26a1, and retinoic acid receptor beta (RARB) are coated with polycomb group (PcG) proteins. Following addition of RA, there is a rapid dissociation of the PcG proteins from the RA target genes.^20^, ^21^ The literature is divided on the exact role of RA signaling in different systems, likely due to the complexity and timing involved in these processes. For example, it has been demonstrated that inhibition of RA signaling can expand hematopoietic stem cells (HSCs), but other studies have shown increased self‐renewal in HSCs with RA addition.^22^, ^23^

In the cardiac area, RA has been shown to increase the number of cardiomyocytes when added during embryonic stem (ES) cell differentiation.^24^ The effects of all‐trans retinoic acid (ATRA) on the expression of RA target genes and on proliferation of cell types present in the heart: endothelial cells, smooth muscle cells (SMCs), cardiac fibroblasts (CFs), and cardiomyocytes were examined, and in all cell types an increase in the expression of RA target genes was seen. ATRA inhibited the proliferation of human aortic SMCs and murine CFs.^25^ There is some evidence that there is a link between RA treatment and the induction of cardiac‐specific genes (myosin light chain [MLC]‐2v, α‐cardiac myosin heavy chain [MHC]), with RA binding RARs and activating early CPCs via NKX2.5, which then promote expression of additional cardiac‐specific genes, accelerating differentiation.^26^, ^27^ In addition, ventricular hypoplasia has been seen after a deficiency of vitamin A, the nonoxidized precursor of RA.^28^ In mouse models, the role of RA signaling in cardiac development and differentiation has been established by knockouts of the RA receptor isoforms.^29^ RA has been well studied with regard to differentiation, both in the ES cell arena as well as in cancer cells. However, RA has multiple physiological effects, including cell proliferation that has not been as well studied in the cardiovascular context and which is of significant interest in the regenerative medicine arena.

In this study, a phenotypic screen was performed to identify compounds, which proliferate human CPCs to enhance their number while maintaining their progenitor phenotype. RAR agonists were identified as potent proliferators of CPCs as measured by nuclear count and their activity and pharmacology confirmed in biochemical and RAR agonist‐antagonist competition experiments. These compounds were subsequently shown to not proliferate CFs, a numerous and critical cell type in the human heart. After treatment with RAR agonists, the CPCs maintained their phenotype as shown by NKX2.5 expression. We subsequently evaluated the effects of RAR agonists on cardiac markers, following differentiation of human pluripotent stem cells (hPSCs), HuES6, from a time point when cardiac progenitor markers were still highly expressed.

This work demonstrates the feasibility of identifying key regulators and biological pathways as well as compound tools by phenotypic screening to study stem cell biology and cardiac regeneration and potentially yield new therapeutic mechanisms to ultimately treat patients.

## 3 MATERIALS AND METHODS

### 3.1 CPC proliferation assay

The CPC proliferation assay including image and data analysis was performed as previously described.^14^ Cardiac progenitor marker antibodies are listed in Table S3.

### 3.2 Compound library screening

#### 3.2.1 *Compounds*

ATRA, retinol, 9‐cis‐retinoic acid, AM580, AM80, and SR11237 were purchased from Sigma‐Aldrich, St. Louis, MO. CD 3254, TTNPB, and EC23 were purchased from Tocris Bioscience, Bristol, U.K. AGN193312 was synthesized over two steps (see Supporting Information). The synthesis of AGN194301 has been described previously.^30^

#### 3.2.2 *Primary screen and hit confirmation*

A library of 10 000 compounds was screened in singlicate at three concentrations (0.1, 1.0, and 10 μM) in the CPC proliferation assay. Each plate contained neutral controls, 0.1% DMSO and maximum controls, 100 ng/mL human basic fibroblast growth factor (FGF; Invitrogen, ThermoFisher Scientific, Waltham, Massachusetts). The data were analyzed using the Genedata Screener software (Genedata, Inc, Basel, Switzerland). A compound was considered active if % proliferation exceeded 3× the SD of the neutral control. Results were calculated using the following calculation method: compound % effect = 100 × [(*X* − min)/(max − min)], where *X* represents a normalized value for the compound based on the neutral and maximum controls.

Active compounds were selected and serially diluted to create a 10‐point concentration range in DMSO.

#### 3.2.3 *CF proliferation assay*

The CF proliferation assay was performed exactly as previously described.^31^

### 3.3 RAR, coactivator recruitment assay

RAR ligand binding domain (LBD)‐SRC1‐2 coactivator peptide (NCOA1_677‐700 biotinylated) recruitment assays were established for the three RAR isotypes RARA (alpha), RARB (beta), and RARG (gamma) to determine relative potencies. The assays were run in white 384‐well plates (Greiner:781075). A total of 0.4 μL of a fixed concentration range of test ligands dissolved in dimethyl sulphoxide (DMSO) was added to assay plates using an acoustic liquid dispenser. Twenty microliters of an assay mix comprising 5 nM 6His tagged LBD, 50 nM biotinylated NCOA1_677‐700, 10 nM Streptavidin‐APC, and 0.375 nM Eu‐W1024 labeled anti‐6xHis antibody in assay buffer (0.02 M Tris, pH 7.5, 0.05 M NaCl, 0.125% CHAPS, 0.05% BSA, and 0.002 M DTT). Plates were incubated at room temperature for 1 hour in the dark, prior to reading in a Pherastar multimode plate reader using homogeneous time resolved fuorescence filter set (ex 320 nm, em 612 nm and 665 nm). The fluorescence resonance energy transfer (FRET) signal at 665 nm was divided by the signal at 612 nm and multiplied by 10 000 to generate a signal ratio value for each well. The raw data were transformed to % effect using the equation: compound % effect = 100 × [(*X* − min)/(max − min)], where *X* represents the effect in the presence of test compound and max and min are the effects in presence of the maximum and minimum controls, respectively.

The data were plotted to generate concentration‐response profiles and the concentration‐response curves were fit to the data using the four‐parameter logistic smart fit method in the Genedata Screener software (Genedata, Inc). To determine activity in antagonist assay format, the compounds were preincubated with the assay mix for 15 minutes then a final 10 nM concentration of RAR agonist (ATRA‐RARA, TTNPB‐RARB, R‐667‐RARG) was added to each well.

### 3.4 RAR agonist‐RAR antagonist competition experiment

Cryopreserved CPCs (Cellular Dynamics International, Madison, Wisconsin) were thawed, plated, and analyzed exactly as described previously.^14^ The start concentration for the agonist AM580 and antagonist AGN194301 was 10 μM and they were serially diluted threefold in DMSO to produce a 10‐point concentration range. AM580 was tested alone and with three different final concentrations of AGN194301 (0.01, 0.1, and 1 μM). AGN194301 was added to the cells directly after AM580. 0.1% DMSO was used as the neutral control and 50 nM (final concentration) of an AstraZeneca compound, which was identified as a potent proliferator of CPCs from the primary screen and with similar efficacy to basic FGF, as the positive/maximal control.

### 3.5 Quantitative polymerase chain reaction

To quantify the mRNA expression of RAR genes, TaqMan Fast Advanced Mastermix (Applied Biosystems/ThermoFisher Scientific, 4444557, Waltham, Massachusetts) was added to 4 ng/μL cDNA together with the appropriate primers/probes. Table S1 lists the primers and probes used for quantification. TaqMan was performed in triplicates on an Applied Biosystems QuantStudio 7 instrument. Data were recorded over 40 polymerase chain reaction (PCR) cycles and the number of cycles required to reach a nominal threshold was measured. Only samples reaching threshold values before cycle 36 were included. The relative expression levels were calculated according to the formula 2^−ΔCt^, where ΔCt is the difference in threshold cycle (Ct) values between the target gene and the ribosomal protein large P0 (RPLP0) endogenous control.

### 3.6 RNA Seq experiment and analysis methods

CPC was seeded in fibronectin‐coated 24‐well plates, 150 000 cells in 0.5 mL media per well. After 24 hours incubation, AM580 and vehicle controls were added to the wells. Cell media were removed after 4 hours or 24 hours, and the cells were lysed for RNA preparation using the MagMax Total RNA Isolation kit. Triplicate samples were prepared for each treatment and time point. RNA integrity and concentration were analyzed on a fragment analyzer with the standard sensitivity RNA kit (Agilent, Santa Clara, California). RNA quality number was 8.2 on average.

A total of 1000 ng RNA per sample was processed with the TruSeq stranded mRNA kit (Illumina, San Diego, California) following the instructions from the manufacturer. QC on amplified libraries was performed on a fragment analyzer with the standard sensitivity NGS kit (Agilent). Samples were pooled, diluted, and denatured according to Illumina's recommendations and sequenced on a NextSeq500 (High 150 cycles kit, reading 2 × 75 cycles). On average, samples had 13M reads with 93% of reads with >Q30.

RNA‐Seq data were processed using the bcbio pipeline version 0.9.7. Hisat2 version 2.0.2‐β aligner was used to align reads to the human genome version hg38. Gene‐level counts were derived using featureCounts version 1.4.4. Differential expression analysis was performed on feature counts using DESeq2.^32^ Data quality was assessed using MultiQC.^33^ Genes with the lowest expression levels (less than five counts in at least two samples) were removed prior to differential expression estimation since they can be considered as nonexpressed.

### 3.7 Directed induction of cardiac progenitors

HuES6 hPSCs^34^ were maintained in defined FTDA medium.^35^ Upon replating, fully confluent cultures into Matrigel‐coated 24‐well plates, cardiac differentiation was induced under serum and albumin‐free conditions at 500 000 cells per well in 2 mL of differentiation medium.^36^ Briefly, cells were treated with 20 ng/mL FGF2, 0.5‐1 ng/mL BMP4 (R&D), and 1 μM CHIR99021 (Axon Medchem) in serum and albumin‐free culture medium composed of knockout‐Dulbecco's modified Eagle's medium (DMEM; ThermoFisher Scientific), 1× insulin/transferrin/selenium supplement (Corning, New York), 10 μM Y‐27632 (Tocris Bioscience, Biotechne, Minneapolis, Minnesota), and penicillin/streptomycin. Insulin and signaling agonists were omitted after 1 day. On days 2‐3, the nascent mesodermal cells were specified to the cardiac lineage using 0.2 μM C‐59 (R&D Tocris) supplementation. Culture medium was replaced daily.

To monitor whether retinoids transiently stabilize the cardiac precursor state, differentiating cells were replated on Matrigel‐coated 24‐well plates on day 5 of differentiation, in the presence of Y‐27632 and at a ratio of 1:24 following Accutase digestion. Basal cardiac progenitor medium was composed of DMEM/F12 (ThermoFisher Scientific), insulin/transferrin/selenium supplement, 0.1% human serum albumin (Biological Industries Inc, Cromwell, Connecticut), 1× chemically defined lipids (ThermoFisher Scientific), and antibiotics. RAR agonists (1 μM) were added from the day of passaging the cells. Samples were harvested and monitored after 4 days of treatment.

### 3.8 HuES6 hPSC quantification, RT‐qPCR, and immunocytochemistry

Cell numbers per well were quantified following single‐cell dissociation with Accutase and using standard Neubauer counting chambers. RT‐qPCR and immunocytochemistry were performed according to standard procedures. Primers validated for high‐efficiency amplification are given in Table S2. RPL37A served as housekeeping gene for normalization. Antibodies used were anti‐ISL1 (DSHB #39‐405, 1:15, and R&D Systems, Minneapolis, Minnesota, #AF1837, 1:100), anti‐CTNT (ThermoFisher Scientific #MS‐295‐P, 1:150), and anti‐NKX2.5 (R&D #AF2444, 1:100).

## 4 RESULTS

### 4.1 Screening for molecules that induce proliferation of iPS‐derived CPCs

A library of 10 000 compounds was screened in our CPC proliferation assay. The library contained representative and structurally diverse molecules from the AstraZeneca corporate collection with activity values (*K*_*i*_, EC_50_ or IC_50_) of less than 100 nM against more than 1600 biological targets representing different classes including GPCRs, nuclear receptors, ion channels, and enzymes. The second component of the screening set included 800 compounds with a reported ability to modulate the phenotype of stem or primary cells. This component included compounds inducing proliferation or differentiation, compounds affecting key developmental pathways (for example, Wnt, TGFβ, Notch, Hedgehog, and Hippo pathways), and compounds acting on epigenetic targets such as bromodomain inhibitors.

**Figure 1:**
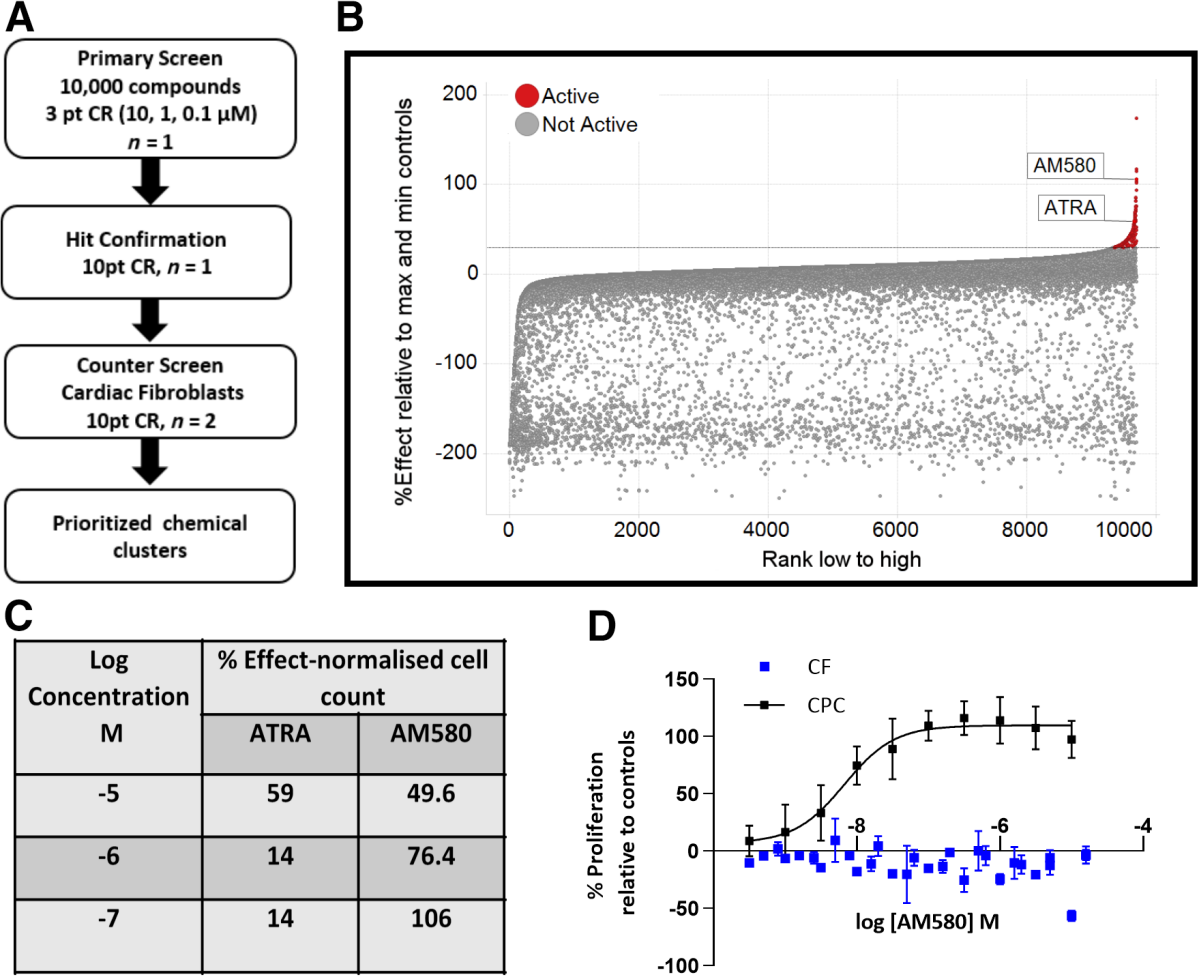
Phenotypic screen identifies AM580 and all‐trans retinoic acid (ATRA) as proliferators of iPS‐derived cardiac progenitor cells. A, Screening cascade for identification and validation of hits. B, ATRA and AM580 were tested at three different concentrations 0.1, 1.0, and 10 μM in the primary screen. The percentage effect is expressed relative to the control, basic FGF at 100 ng/mL. C, Percentage effect relative to max and min controls versus compounds ranked according to the highest effect at any concentration for each compound. D, AM580 concentration response effects on the proliferation of cardiac progenitor cells (CPCs) and cardiac fibroblasts (CFs) measured as nuclei count, normalized to % effect from the maximal and neutral controls for both CPCs and CFs. Data represent the mean of at least three independent experiments where each experiment consisted of technical duplicates or triplicates, with error bars = SEM

The library was screened in three‐point concentration response (CR) at 0.1, 1.0, and 10 μM measuring proliferation of human induced pluripotent stem (iPS)‐derived CPCs with nuclear count, as measured by Hoechst staining, as the primary endpoint. The screening cascade outlining the progression of hits is described in Figure 1A. We classified compounds as hits if they induced a greater than 30% increase in cell count relative to the positive control bFGF at 100 ng/mL. The hit rate at three concentrations was 3.5%. Figure 1B summarizes the normalized data for all compounds. The entire screening set could be clustered using extended connectivity fingerprints giving 2540 unique clusters, of which 1026 were singletons. Compounds classified as active were distributed over 223 individual clusters or singletons.

Evaluating both potency and efficacy led to the identification of two RAR agonists, namely ATRA and AM580^37^, which increased the number of CPCs. AM580, at 0.1 μM, proliferated the cells with comparable efficacy to 100 ng/mL bFGF (Figure 1C), which was 1.6‐fold greater than unstimulated controls. To validate both compounds as hits from the primary phenotypic screen, they were followed up in 10‐point concentration response testing using the same assay, which confirmed that ATRA and AM580 were very potent and efficacious drivers of proliferation of the iPS‐derived CPCs, where AM580 was ~10‐fold more potent than ATRA (EC_50_ values 5 nM and 56 nM, respectively). Although the efficacy of AM580 did not reach 100% at the top concentrations in the primary screen, purified batches of AM580 showed a consistent and robust potency and efficacy in the 10‐point CR across occasions and using different batches of CPCs. In contrast, the results with ATRA were far more variable between occasions ranging from an EC_50_ of 50 nM to a value >10 μM. This is likely due to the known instability of the molecule toward isomerization across the trans‐double bonds, which can occur when exposed to light in the laboratory.^38^

To ensure that these molecules were not general proliferators of all cardiac cells, they were also tested in a human CF proliferation assay^31^ using nuclear count as the endpoint. Both compounds were inactive on CFs and the data for AM580 in comparison to proliferation of CPCs is shown in Figure 1D.

### 4.2 Expanded CPCs maintain their progenitor phenotype

**Figure 2:**
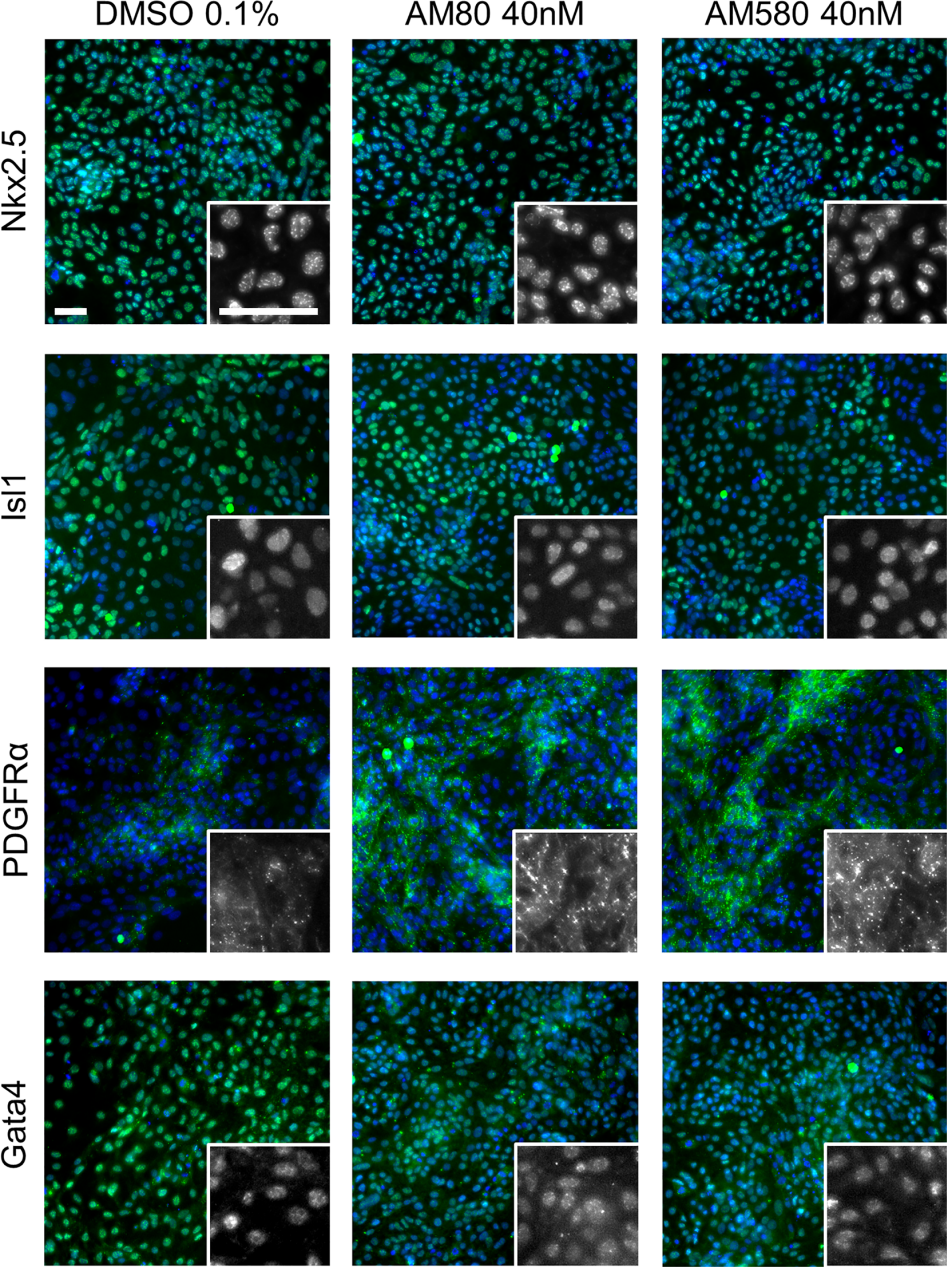
Cardiac progenitor cells (CPCs) retain progenitor markers after retinoic acid receptor agonist treatment. Representative images from wells where CPCs were treated for 3 days with 0.1% DMSO, 40 nM AM580, or 40 nM AM80 and then stained for cardiac progenitor markers Nkx2.5, Islet1, Gata4, and PDGFRα. Larger images are representative merged images of Hoechst nuclear stain in blue and immunofluorescence of the indicated progenitor marker in green. Insets show the progenitor marker staining in gray scale at 3.3‐fold higher magnification. Scale bars: 50 μm

A key requirement of the screen was that the expanded CPCs must retain their progenitor phenotype. CPCs were treated with two RAR agonists, AM580 and AM80, for 3 days before antibody staining for the cardiac progenitor markers Nkx2.5, Islet1(Isl1), Gata4, and PDGFRα. Both AM580 and AM80‐treated cells retain expression of these four key nuclear and membrane located markers of CPCs (Figure 2).

### 4.3 Confirmation of RAR agonists driving the proliferation of the CPCs

ATRA is known to isomerize to 9‐cis‐retinoic acid (9‐cis RA) which also binds to RXR.^39^, ^40^ On the other hand, AM580 is an analogue of RA that does not undergo isomerization and is therefore selective for RAR over RXR. The stereoisomeric compound 9‐cis RA^39^ was inactive in the CPC proliferation assay, showing that isomerization of this compound to ATRA in this cell system was either not occurring or occurring too slowly to affect the proliferation of the cells. Additional RXR agonists such as CD3254^41^ and SR11237^42^ were also tested and these were inactive suggesting that the proliferation phenotype is driven by activation of RAR. To increase the evidence that RAR agonism drives the phenotypic effect of increased cell proliferation, additional RAR agonists were tested in the CPC assay, including AM80,^43^ AGN193312,^44^ TTNPB,^45^ and EC23.^46^ All of these compounds caused potent and robust proliferation of the CPCs with EC_50_ values in the low nanomolar range and with similar efficacy compared with AM580 (Table 1). To investigate the structure‐activity relationship, retinol as a precursor of ATRA was tested and showed no effect on the proliferation of the cells. Retinol was also inactive in the RAR coactivator recruitment assay (Table 1).

**Table 1:** RAR agonists are potent proliferators of iPSC‐derived CPC

Compound	Structure	CPC pEC_50_	RARα pEC_50_ (% top effect)	RARβ pEC_50_ (% top effect)	RARγ pEC_50_ (% top effect)	RARα pIC_50_ (% top effect)	RARβ pIC_50_ (% top effect)	RARγ pIC_50_ (% top effect)
ATRA	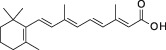	7.3 ± 0.3	8.1 ± 0.3 (103)	8.3 ± 0.3 (104)	8.2 ± 0.2 (104)	—	—	—
Retinol	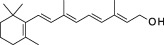	<5.0	<4.7	<4.7	<4.7	<4.7	<4.7	<4.7
9‐cis‐retinoic acid	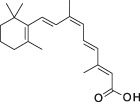	<5.0	<4.7	<4.7	<4.7	<4.7	<4.7	<4.7
AM580	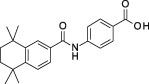	8.2 ± 0.3	8.4 ± 0.1 (157)	8.1 ± 0.1 (104)	7.2 ± 0.1 (99)	<4.7	<4.7	<4.7
AGN 193312	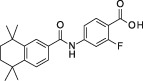	8.1 ± 0.3	8.4 ± 0.1 (159)	8.0 ± 0.1 (100)	7.0 ± 0.1 (88)	—	—	—
AM80	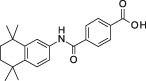	8.1 ± 0.2	8.4 ± 0.2 (175)	8.0 ± 0.1 (149)	6.9 ± 0.1 (119)	<4.7	<4.7	<4.7
TTNPB	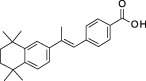	8.5 ± 0.1	8.2 ± 0.1 (193)	8.4 ± 0.2 (181)	8.4 ± 0.1 (134)	<4.7	<4.7	<4.7
EC 23	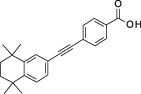	8.9 ± 0.2	8.3 ± 0.1 (172)	8.5 ± 0.2 (144)	8.5 ± 0.1 (135)	<4.7	<4.7	<4.7
CD 3254	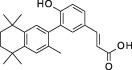	<5.0	<4.7	<4.7	<4.7	<4.7	<4.7	<4.7
SR 11237	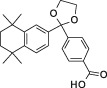	<5.0	<4.7	<4.7	<4.7	<4.7	<4.7	4.9 ± 0.1 (59)^a^
AGN 194301	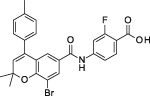	<5.0	<4.7	<4.7	<4.7	8.6 ± 0.4 (108)	8.9 ± 0.1 (105)	7.7 ± 0.2 (106)

Abbreviations: ATRA, all‐trans retinoic acid; CPC, cardiac progenitor cell; iPSC, induced pluripotent stem cell; RAR, retinoic acid receptor.

**Figure 3:**
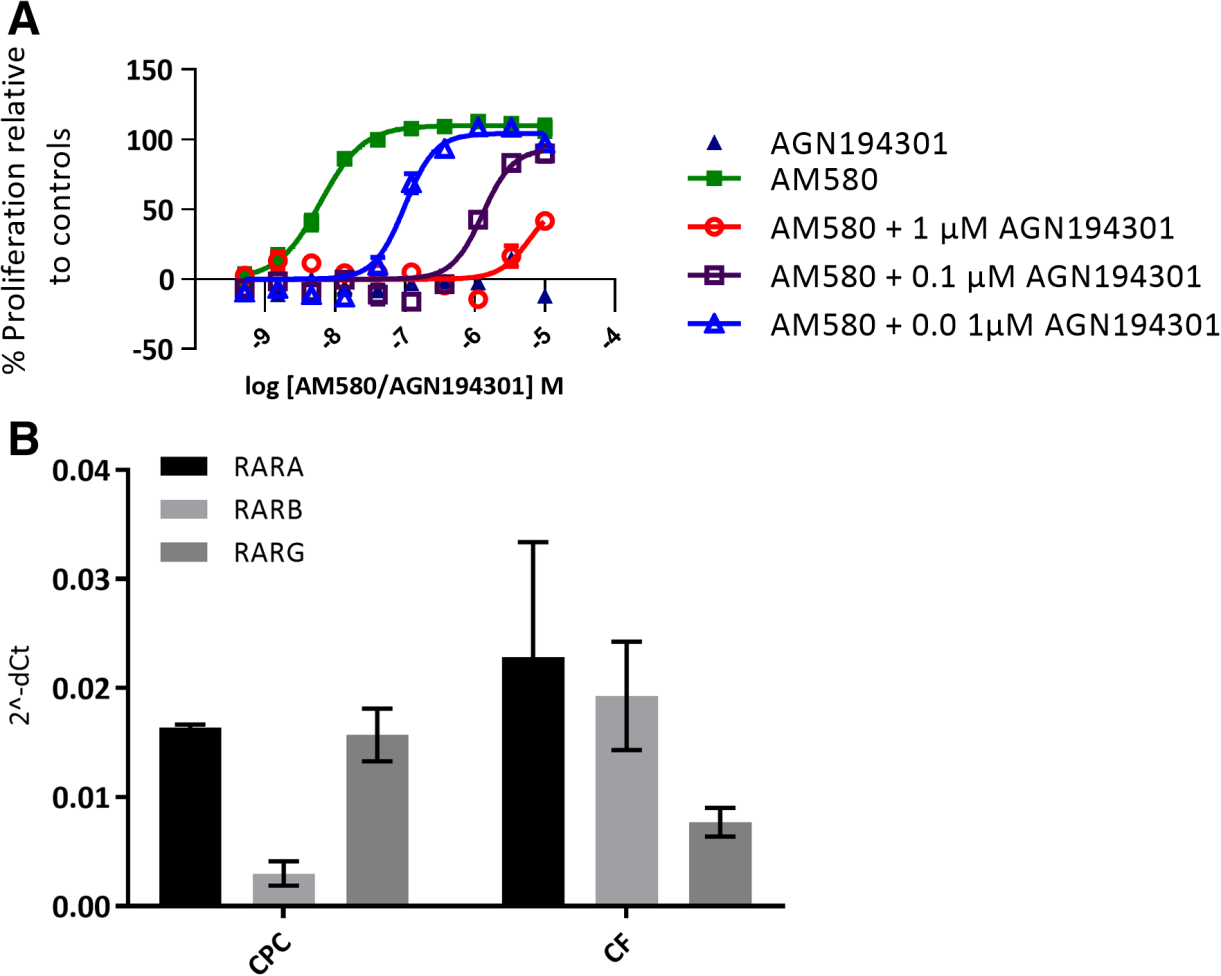
A, Effect of a competitive retinoic acid receptor (RAR) antagonist AGN194301 in three different concentrations on RAR agonist AM580‐induced cardiac progenitor cell (CPC) proliferation, 10‐point concentration response curve. Cells were fixed and nuclei stained on day 4 postplating and 3 days postcompound addition. Proliferation was measured as nuclei count normalized to % effect from the maximal and neutral controls. pEC_50_ for AM580 is attenuated by adding increased concentrations of AGN194301, pEC_50_ shifts from 8.2 for AM580 to pEC_50_ 7.0 when 0.01 μM AGN194301 is present and to 5.9 with 0.1 μM and to 5.5 with 1 μM of AGN194301. There was no effect on proliferation by AGN194301 alone. The data represent the mean of two independent experiments each conducted in quadruplicate; error bars indicate SEM. B, RARs are expressed in iPS‐derived CPCs and cardiac fibroblasts (CFs). The data for CPCs and CFs are obtained from two independent RNA preparations from each cell type

To provide further evidence for the mode of action of RAR activation, a pharmacological competition experiment was performed (Figure 3A). Here, the concentration response of the RAR agonist AM580 in the CPC proliferation assay was run in the presence of three different concentrations of the RAR competitive antagonist, AGN194301.^30^ The proliferative effect of the RAR agonist AM580 was attenuated with increasing concentrations of the antagonist. The RAR antagonist, in the absence of RAR agonist, did not proliferate the cells and showed no signs of toxicity.

To confirm the presence of the RA receptors in the cells, the expression of the three RAR isotypes RARA, RARB, and RARG was confirmed in untreated iPS‐derived CPCs and CFs by Taqman gene expression analysis (Figure 3B). The data highlight that the RARs are present in this cell type that a range of RAR agonists cause potent proliferation of the cells and that the effect of a RAR agonist can be attenuated in the presence of a RAR antagonist, which taken together, support a conclusion that agonism of RAR drives the proliferative effect observed in the iPS‐derived CPCs.

AM580 has been reported to be selective for activating RARA.^37^ However, we observed on average a 0.3 log unit difference in potency with respect to RARB and one log unit with respect to RARG while testing in RAR FRET‐based NCOA1_677‐700 coactivator peptide recruitment assays (Table 1). The RAR coactivator peptide recruitment assay provides a robust method to screen and directly compare the potencies of RAR ligands as agonists or antagonists of peptide recruitment versus the three RAR isotypes. The data showed that the RAR agonist compounds tested were highly potent on RARA and RARB and either equipotent across the RAR isoforms or with up to 10‐fold selectivity toward RARG. All RAR agonist compounds exhibited high potency at proliferating CPCs and this study could not distinguish the isotype specificity required for CPC proliferation.

### 4.4 AM580 affects the expression of RAR target genes and cardiac cell markers in CPCs

**Figure 4:**
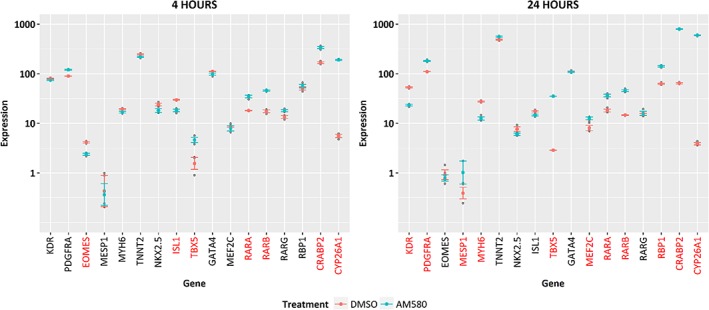
RNA seq analysis of AM580‐treated cardiac progenitor cells (CPCs). Expression levels are shown as logarithm base 10 of transcripts per kilobase million for DMSO and AM580‐treated CPC samples, for selected marker genes at 4 hours and 24 hours post‐treatment. Genes with expression levels significantly different (*P* < .05) between treatment and control are marked in red font. Significance was determined using an analysis of variance model for treatment, time, and gene followed by Tukey's correction for multiple testing

RNA seq analysis was performed on CPCs incubated with AM580 for 4 hours and 24 hours (Figure 4). CPC samples express mRNA for all three RAR subtypes (confirming what we have seen in the Taqman experiment highlighted above) and as expected, upon treatment with AM580 a significant increase in mRNA expression was observed for several well‐characterized RAR‐responsive genes (RARB, RBP1, CRABP2, and CYP26A1). This was observed at both 4‐hour and 24‐hour time points and is consistent with the hypothesis of AM580 acting to induce RAR‐mediated transcription in this cell type.

Early cardiac markers eomesodermin (EOMES) and mesoderm posterior 1 (MESP1 BHLH transcription factor) were still detected, albeit at very low signal levels. Cardiac progenitor markers NKX2.5, GATA4, MEF2C, TBX5, ISL, KDR, and PDGFRA mRNA were expressed in the CPC samples at both time points, with some showing modulation by AM580 at the different time points. KDR, PDGFR‐α, and NKX2.5, the initial three markers of the CPC population are still significantly expressed after RAR agonist treatment which provides additional support for the maintenance of the progenitor phenotype of the cells. Interestingly, cardiac troponin T (TNNT2) and MYH6 expressions were also noted with the latter decreasing upon AM580 treatment. This may be indicative of presence of a minor subpopulation of cells with differentiated cardiomyocyte characteristics.

### 4.5 RAR agonists universally antagonize terminal differentiation in human cardiac precursors

**Figure 5:**
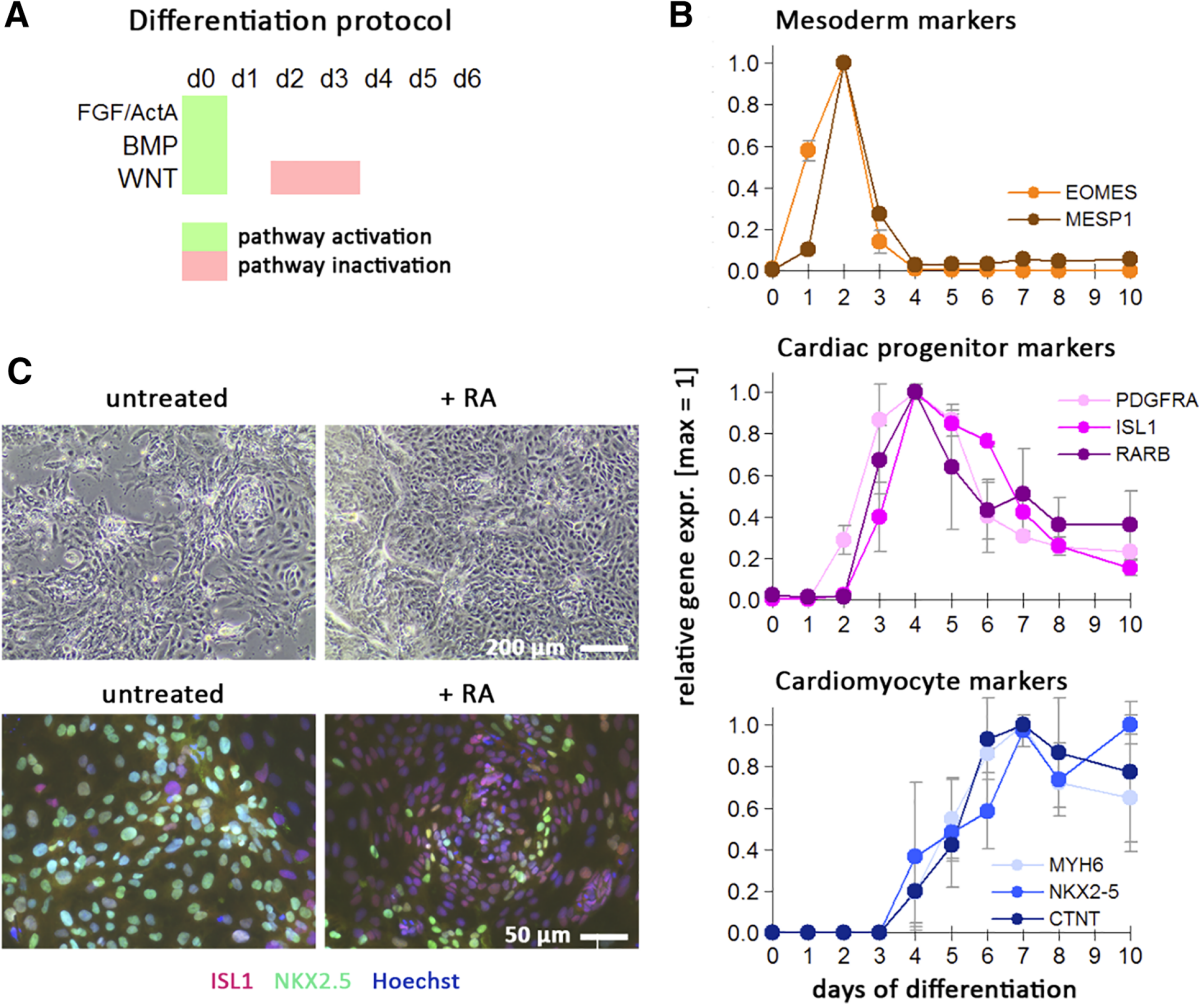
Retinoic acid (RA) counteracts terminal cardiac differentiation in transient progenitor cells. A, Schematic of cardiac induction protocol. Upon replating undifferentiated human pluripotent stem cells at high density, the indicated signaling pathways were simultaneously activated for 1 day, followed by WNT inhibition on days 2‐3. Spontaneously beating monolayers of immature cardiomyocytes are typically obtained after 6‐7 days. B, Characterization of cardiac induction time‐course using markers of intermediate stages (mesoderm, cardiac progenitors) and early cardiomyocytes. Data are from two independent experiments analyzed by RT‐qPCR and microarrays (merged data normalized to peak expression ± SEM). C, Cardiac progenitors replated on day 5 of differentiation and exposed for another 4 days to baseline conditions or RA (1 μM). The majority of control cells differentiates further into NKX2.5‐positive cardiomoyctes and display spontaneous beating. RA‐treated cells show an increase in cell numbers, compromised NKX2.5 induction, and sustained ISL1 expression in most cells

Next, we sought to assess whether these findings may have broad validity by monitoring the effects of RA agonists on human cardiac progenitors from an independent source and from a different genetic background. To this end, human embryonic stem cells (hESCs) HuES6 were subjected to cardiac induction using a signaling perturbation procedure yielding a near‐homogeneous population of CPCs (Figure 5A).^36^, ^47^ Using this protocol, hESCs transitioned through expected intermediate stages of cardiac development, namely mesoderm, marked by EOMES and MESP1, as well as a transient cardiac progenitor state marked by PDGFRA, ISL1, and interestingly, the RAR gene RARB (Figure 5B). The latter observation may point toward a RARβ‐mediated retinoid responsiveness of the cells particularly around day 4 of differentiation and may maintain the progenitor phenotype.

Independent experiments using day‐4 differentiating cells, however, confirmed that RA also acts as a potent inducer of an atrial cell fate at this stage,^48^ but this was not seen using day‐5 cells.^49^ Hence, we focused our efforts on cells isolated on day 5 of cardiac induction, a time point when cardiac progenitor markers (PDGFRA and ISL1) were still highly expressed, whereas terminal differentiation markers were still below maximum levels (Figure 5B). Day‐5 differentiating hPSCs were replated at a low seeding density and the majority of these cells continued to differentiate into cardiomyocytes, as they displayed NKX2.5 induction and spontaneous beating 4 days later (Figure 5C, left). By contrast, cells at day 5 treated with RA (1 μM) did not acquire a beating phenotype, were mostly negative for NKX2.5, and displayed persistent staining for ISL1 at the protein level (Figure 5C, right). These data indicate that RA signaling inhibits terminal cardiac differentiation in the transient human cardiac precursor state and may maintain the progenitor phenotype.

**Figure 6:**
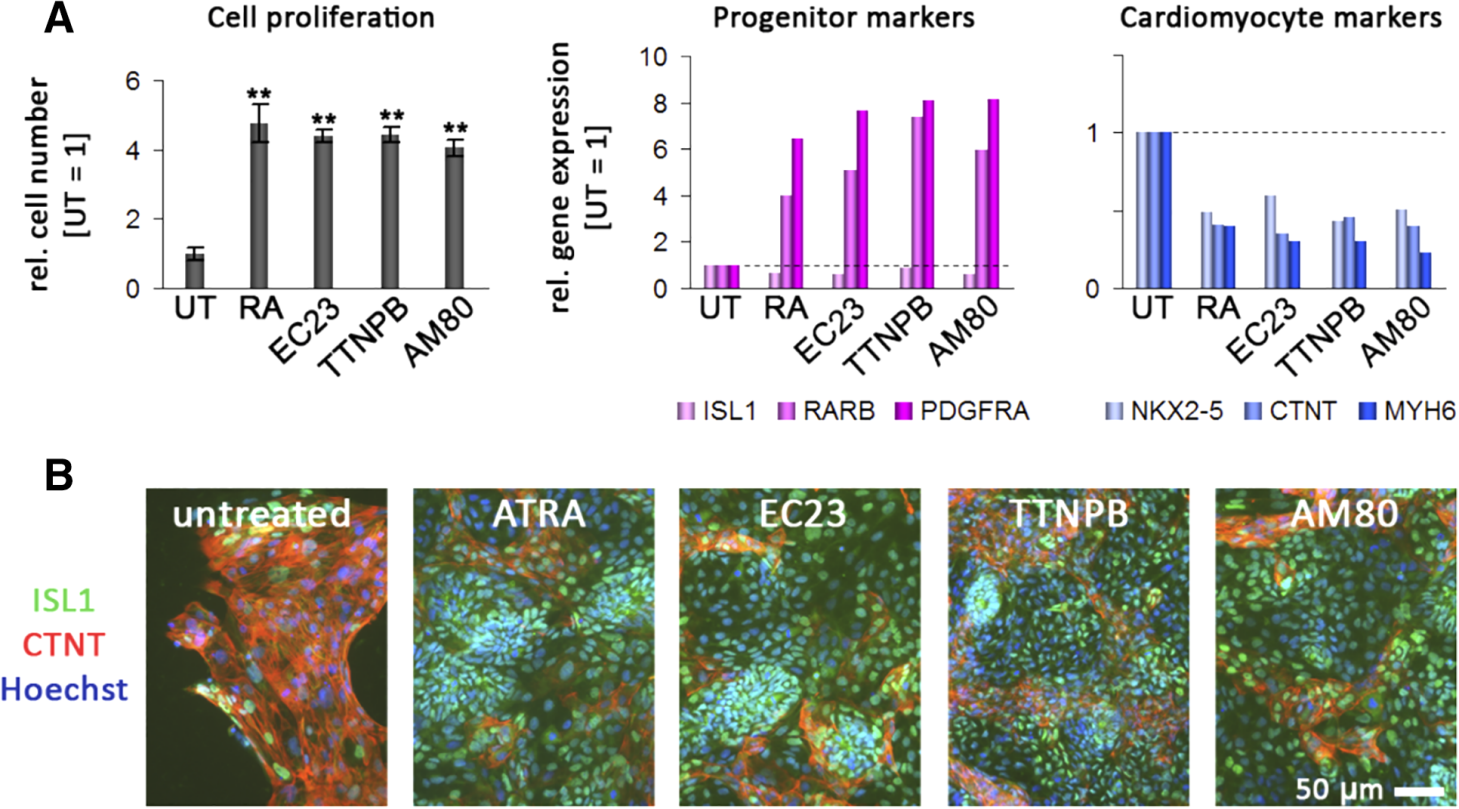
Effects of various retinoic acid receptor (RAR) agonists on proliferation and expression in cardiac progenitors derived from differentiated human pluripotent stem cells. A, Effects on proliferation and gene expression on replated day‐5 progenitors following 4 days of exposure to the indicated compounds. All‐trans retinoic acid (ATRA), EC23, TTNPB, and AM80 (all at 1.0 μM) had strong positive effects on cell proliferation (left panel, n = 3, *P* < .01, pairwise against untreated cells). Note the overall positive association between cell proliferation and cardiac precursor gene expression, and the negative relationship between cell numbers and cardiomyocyte marker induction (plots on the right). B, Immunocytochemistry analysis of ISL1 and cardiac troponin T (CTNT) in the same samples. Note again the increase in cell numbers, sustained ISL1 expression at the protein level, and inhibited CTNT induction with ATRA, EC23, TTNPB, and AM80

To assess whether this observation reflects a general feature of RA agonists, we next exposed day‐5 replated cells to a selection of the previously studied set of compounds, namely RA, EC23, TTNPB, and AM80. Compared with untreated samples, all these molecules enhanced the expression of cardiac progenitor markers with the exception of ISL1. Terminal cardiac differentiation was inhibited by some of these molecules, whereas proliferation rates were stimulated beyond basal levels (Figure 6A, left panels). All the RA agonists tested resulted in a fourfold to fivefold relative increase in progenitor cell numbers within 4 days (Figure 6A, top panel).

Interestingly, the increase in cell proliferation across the different compounds was positively correlated with the increase in cardiac precursor gene expression and conversely, there was an antagonistic correlation with terminal cardiomyocyte differentiation (Figure 6B). These data were further validated at the protein level following 4 days of exposure to the compounds. RA, EC23, TTNPB, and AM80 again caused a pronounced inhibition of terminal cardiac differentiation, whereas most cells remained ISL1‐positive within the time frame of the assay (Figure 6B). Taken together, these data indicate that RA agonists, at least transiently, enhance proliferation of human cardiac precursor cells in general, at the expense of terminal cardiac differentiation. It remains to be investigated whether this finding also bears potential for the long‐term stabilization and expansion of these precursors.

## 5 DISCUSSION

Identification of new therapeutic targets to repair an adult heart after a myocardial infarction is sorely needed and cardiac regeneration would represent a new treatment paradigm. Progenitor cells exist in the adult human heart that can play a role in cardiac repair via paracrine mechanisms and direct contribution to vasculature formation.^15^ However, these cells are rare and difficult to isolate and therefore little is known about the molecular mechanisms and signaling pathways required for their proliferation and differentiation. In the absence of adult human cells for testing, human iPS‐derived cells can be used as a model system to investigate and discover novel biology in the cell type of interest. Herein, we describe the use of our previously reported human iPS‐derived CPCs in a phenotypic screening campaign to identify molecules, which enhance the proliferation and expand the population of these cells. This approach enhances our understanding of the key pathways driving cell cycle activity and differentiation capability of CPCs and can be used to identify potential therapeutic targets for cardiac regeneration.

Screening a compound library of 10 000 compounds led to the identification of RAR agonists as potent and robust proliferators of iPS‐derived CPCs. The nuclear receptor‐ligand‐dependent coactivator recruitment assay used in this study allowed us to rapidly compare EC_50_ data from a selection of known RAR and RXR ligands based on their ability to modulate the affinity of the target RAR or RXR LBD for the labeled coactivator peptide, SRC1‐2. A range of structurally diverse RAR agonists with confirmed activity against the RAR isotypes were shown to proliferate this cell type while showing no proliferation of CFs. In addition, agonists of RXR did not proliferate the cells. The data did not allow us to draw conclusions with respect to the requirement for ligand activity against selected RAR isotypes in order to drive proliferation. The presence of the three RAR isotypes, RARA, RARB, and RARG, in the iPS‐derived cells was confirmed and the proliferative effect of the RAR agonists could be competed away through treatment with a RAR antagonist, supporting that the proliferative effect was indeed driven by agonism of the RA receptor. Treatment of the iPS‐derived CPCs led to a significant increase in mRNA expression for several well‐characterized RAR‐responsive genes as well as cardiac progenitor markers being expressed in the CPC samples at 24 hours after treatment.

CPCs can be obtained from different sources and genetic backgrounds and here we show that the identified RAR agonists also proliferate a separate CPC population derived from hPSCs, HuES6, but did not proliferate CFs, demonstrating that the effects of RAR agonism may be specific to certain cell populations in the myocardium. With the HuES6 cells, the RAR agonists had a time‐of‐differentiation‐dependent effect on the cells. These data indicate that RAR agonists can proliferate cardiac precursor cells in general and maintain/increase the levels of progenitor markers during treatment, preventing terminal cardiac differentiation.

The impact of RA signaling on the differentiation and proliferation of cardiac cells is dependent on the cell type and the differentiation status of the cells, and it is known that RA plays many critical roles during development, including acting as a morphogen, inducing commitment of precursor cells and thereby mediating tissue patterning.^13^, ^14^ One of the first studies of RA signaling in the developing mouse heart revealed key regulatory functions for RA signaling in chamber development and specification.^50^ In addition, research in zebrafish and mouse both indicated that RA signaling restricts the pool of cardiac progenitors from multipotent precursor cells.^51^, ^52^ Furthermore, using mouse models, the role of RA signaling in cardiac development and differentiation has been established by knockouts of the RA receptor isoforms where RAR double mutant mice developed abnormalities in the heart as well as other organs.^29^ The effects of ATRA on the expression of RA target genes and on proliferation of certain cell types isolated from the mouse heart has also been reported. In these murine cell types, an increase in the expression of RA target genes was seen while ATRA inhibited the proliferation of human aortic SMC and murine CFs and promoted the proliferation of human umbilical vein endothelial cells (HUVECs)^25^ again indicating the cell type specific effect of RAR agonism. In terms of differentiation, RA has been shown to increase the number of cardiomyocytes when added during ES cell differentiation.^20^ There is also some evidence for a link between RA treatment and the induction of cardiac‐specific genes (MLC‐2v, α‐cardiac MHC), with RA binding RARs and activating early CPCs via NKX2.5, which then promote expression of additional cardiac‐specific genes, accelerating differentiation.^22^, ^23^ Deficiency of vitamin A, the nonoxidized precursor of RA has also been shown to lead to ventricular hypoplasia.^24^

Modulation of RA signaling has also been shown to control atrial versus ventricular differentiation from hESCs. Addition of a RA receptor antagonist to differentiating cell cultures gave mainly ventricular‐like cells, whereas addition of RA yielded atrial‐like enriched CMs.^53^ This result is in accordance with our data where treatment of day 4 differentiating hPSCs cells induced an atrial cell fate. However, this was not seen with day 5 differentiating hPSCs, where instead proliferation of the progenitor cells at this stage was enhanced at the expense of terminal cell differentiation highlighting the stage of differentiation effects of RA signaling on the cells. Hence, the clearly defined populations of CPCs utilized in this study are at a stage of differentiation where RA signaling leads to proliferation of the cells in contrast to other reported precursor cells where RA signaling leads to cell differentiation. RA signaling in different cell types is highly complex and context‐dependent. In many cell types, RA signaling affects the differentiation of the cells. However, as shown here with both populations of CPCs used, as well as other cell types including hPSCs^54^ and HUVECs described above, RA signaling leads to cell proliferation. Subsequent single‐cell RNAseq or use of spatial transcriptomics could be instructive in further refining cell type‐specific responses to RAR agonism and unraveling the complex role in CPC proliferation and differentiation, beyond the bulk cell‐population approach used in this study.

Outside of the cardiac area, RA has also been described to proliferate and differentiate a variety of cell types including various cancer cell types and progenitor cells of the central nervous system including glial and neural progenitor cells. In this setting, RA signaling has been shown to promote neurogenesis from stem cells in a chick model system.^55^ However, as also shown in our study, the effects of RA signaling are context‐dependent leading to different effects on neuronal differentiation and proliferation. For example, it has recently been reported that RA is required to promote proliferation of neural stem and progenitor cells in the adult hippocampus.^56^ Combined with the results of the HuES6 cells described herein where there was a time‐of‐differentiation‐dependent effect, again implies that the differentiation status of the cells plays a large role in how the cells respond to RA treatment.

Although RA has multiple physiological effects, including cell proliferation, it has not been as well studied in the cardiovascular context and which is of significant interest in the regenerative medicine arena. The work described here highlights the power of phenotypic screening to identify novel molecules and mechanisms that impart the desired phenotype in a relevant cell system. A novel role of RA signaling and RAR agonists has been revealed as an important driver of CPC proliferation to enhance their population and has been shown in two separate CPC populations from an independent source and from a different genetic background. The stage‐specific effects of RA signaling has also been shown in this second population of CPCs where treatment with RA after either 4 or 5 days of differentiation either led to atrial cell differentiation or enhanced proliferation and repression of cardiac differentiation, respectively. In addition, the cell type‐specific effects were also highlighted as CFs did not undergo proliferation.

How this mechanism translates in vivo to activate endogenous CPCs and whether proliferation of these rare cells is sufficient to enhance cardiac repair warrants further study as we continue to identify potential mechanisms that can be targeted to enhance cardiac regeneration.

## 6 CONCLUSION

The data presented here indicate that RAR agonists, at least transiently, enhance proliferation of human cardiac precursor cells in general, at the expense of terminal cardiac differentiation, and may have implications for cardiac regeneration. It remains to be investigated whether this finding also bears potential for the long‐term stabilization and expansion of these precursors.

## CONFLICT OF INTEREST

L.D. is an employee of UCB Pharma; J.M., A.N., S.P., U.K., S.M., E.M., A.D., S.K., I.B., J.S., B.M., and Q.‐D.W. are employees of AstraZeneca; A.T.P. is an employee of Sanofi; B.G. is an employee of the Max Planck Institute for Molecular Biomedicine.

## 7 AUTHOR CONTRIBUTIONS

L.D., J.M., A.N., B.G., A.T.P.: conception and design, collection and assembly of data, data analysis and interpretation, manuscript writing, final approval of manuscript; S.P., U.K., S.M., E.M., A.D., S.K., J.S., B.M.: collection and assembly of data, data analysis and interpretation, final approval of manuscript; I.B.: collection and assembly of data, data analysis and interpretation, manuscript writing, final approval of manuscript; Q.‐D.W.: conception and design, data analysis and interpretation, manuscript writing, final approval of manuscript.

8

## Supporting information


**Data S1**: Supporting Information.

## Data Availability

The data that support the findings of this study are available from the corresponding author upon reasonable request.
