# Supplementary material for: Discovery of retinoic acid receptor agonists as proliferators of cardiac progenitor cells through a phenotypic screening approach
Source: Stem Cells Transl Med. 2019 Sep 11;9(1):47–60. doi: 10.1002/sctm.19-0069 (PMC6954720; doi:10.1002/sctm.19-0069)
Supplement: Supplementary file 1 — Data S1: Supporting Information. [file SCT3-9-47-s001.pdf]

SUPPLEMENTARY INFORMATION

Discovery of Retinoic Acid Receptor Agonists as Proliferators of Cardiac Progenitor Cells Through a Phenotypic Screening Approach

Lauren Drowley [a], Jane McPheat [b], Anneli Nordqvist [a], Samantha Peel [c], Ulla Karlsson [b], Sofia Martinsson [a], Erik Müllers [a], Anita Dellsén [b], Sinead Knight [c], Ian Barrett [c], José Sánchez [b], Björn Magnusson [b], Boris Greber [d], Qing-Dong Wang [a], Alleyn T. Plowright [a]

[a] Research and Early Development, Cardiovascular, Renal and Metabolism, BioPharmaceuticals R&D and [b] Discovery Sciences, R&D, AstraZeneca, Gothenburg, Sweden; [c] Discovery Sciences, R&D, AstraZeneca, Cambridge, United Kingdom; [d] Human Stem Cell Pluripotency Laboratory, Max Planck Institute for Molecular Biomedicine, Münster, Germany

Synthesis of 2-Fluoro-4-(5,5,8,8-tetramethyl-5,6,7,8-tetrahydronaphthalene-2-carboxamido)benzoic acid (AGN193312)

CDI (220 mg, 1.36 mmol) was added to 5,5,8,8-tetramethyl-5,6,7,8-tetrahydronaphthalene-2-carboxylic acid (300 mg, 1.29 mmol) in THF (10 mL) under nitrogen. The resulting suspension was stirred at 30 °C for 3 h. Then methyl 4-amino-2-fluorobenzoate (240 mg, 1.42 mmol) and DBU (0.2 mL, 1 mmol) were added and the suspension was stirred at 80 °C for a further 16 h. The reaction mixture was concentrated and diluted with EtOAc (25 mL), and washed sequentially with 2M HCl (2×25 mL), 2M NaOH (1×25 mL), and saturated brine (3×25 mL). The organic layer was dried over Na_2SO_4, filtered and evaporated to afford crude product which was used directly in the next step.

NaOH (365 mg, 9.13 mmol) in water (4 mL) was added to methyl 2-fluoro-4-(5,5,8,8-tetramethyl-5,6,7,8-tetrahydronaphthalene-2-carboxamido)benzoate (0.35 g, 0.91 mmol) in THF (8 mL) and MeOH (8 mL). The resulting suspension was stirred at 25 °C for 16 h. The

reaction mixture was concentrated and diluted with water (25 mL), the mixture was adjusted to pH 6 with 2M HCl and extracted with EtOAc (3×25 mL), the organic layer was dried over Na_2SO_4, filtered and evaporated to afford pale yellow solid. The crude product was purified by preparative HPLC (XBridge Prep C18 OBD column, 5µ silica, 19 mm diameter, 150 mm length), using decreasingly polar mixtures of water (containing 0.1% Formic acid) and MeCN as eluents. Fractions containing the desired compound were evaporated to dryness to afford the title compound (0.30 g, 63%) as a white solid. ^{1}H NMR (400 MHz, DMSO-d_6) δ 1.29 (d, *J* = 10.8 Hz, 12H), 1.68 (s, 4H), 7.50 (d, *J* = 8.4 Hz, 1H), 7.59 – 7.74 (m, 2H), 7.81 – 7.92 (m, 3H), 10.51 (s, 1H), 12.98 (s, 1H). ^{19}F NMR (400 MHz, DMSO-d_6) δ -108.146 (s, 1F). HRMS (ESI-TOF) m/z $[M+H]^+$ Calcd for $C_{22}H_{24}FNO_3$ 370.1818; Found 370.1816.

The synthesis of AGN194301 has been described previously by Teng et al. [1] ^{1}H NMR (600 MHz, $CDCl_3$) δ 1.56 (s, 6H), 2.39 (s, 3H), 5.70 (s, 1H), 7.18 – 7.27 (m, 5H), 7.49 (d, *J* = 2.1 Hz, 1H), 7.71 (dd, *J* = 1.9, 12.9 Hz, 1H), 7.81 – 7.88 (m, 1H), 7.90 (d, *J* = 2.1 Hz, 1H), 7.98 (t, *J* = 8.4, Hz, 1H). ^{13}C NMR (151 MHz, $CDCl_3$) δ 21.39, 28.04, 78.70, 108.06 (d, *J* = 28.0 Hz), 111.59, 112.75 (d, *J* = 9.4 Hz), 114.82 (d, *J* = 3.0 Hz), 124.07, 124.14, 127.02, 128.55, 129.61, 130.35, 131.58, 133.68, 133.88, 134.20, 138.44, 144.45 (d, *J* = 11.9 Hz), 154.17, 163.45 (d, *J* = 260.7 Hz), 164.39, 167.93.

References

1. Teng M, Duong TT, Johnson AT et al. Identification of Highly Potent Retinoic Acid Receptor α-Selective Antagonists. Journal of Medicinal Chemistry. 1997;40:2445-2451.

Supplementary information Table 1 TaqMan® gene expression assays and primers/probe used for Real-time quantitative PCR.

TaqMan® gene expression assays were purchased from Applied Biosystems/ThermoFisher Scientific and primers/probe were purchased from Sigma.

Gene	Assay (FAM-MGB)	Forward primer (5´-3´)	Reverse primer (5´-3´)	Probe (FAM-TAMRA)
RARA	Hs00940446			
RARB	Hs00977140			
RARG	Hs01559234			
RPLP0		CCATTCTATCATCAACGGGTACA	AGCAAGTGGGAAGGTGTAATCC	TCTCCACAGACAAGGCCAGGACTCGT

Supplementary information Table 2 Primers used for RT-qPCR of hPSCs. RPL37A served as housekeeping gene for normalization.

**Gene**	**Forward primer (5´-3´)**	**Reverse primer (5´-3´)**
EOMES	CTTGCTAGGCCTCTGCTGTGTG	TTGGTGACTCCTTAGCTTGCTCTCT
CTNT	GGCAGCTCCTGTTTGGAAATG	TTATTACTGGTGTGGAGTGGGTGTG
ISL1	TTTATTGTCGGAAGACTTGCCACTT	TCAAAGACCACCGTACAACCTTTATCT
MESP1	CAACTGACGCCGTCTCTGTGA	GTCTGCCAAGGAACCACTTCG
MYH6	ACCTGGTGGACAAGCTGCAA	CACCTTGCGGAACTTGGACA
NKX2.5	ACCGATCCCACCTCAACAGC	CTCCGCAGGAGTGAATGCAA
PDGFRA	TTGCTGTGAGCCTTGCATGA	GTGGGAGCATTTGTTAGGACTGG
RARB	TGCTCTTTCTGATGCTCTCAAACTG	TCTTCTGCTAGTGCAGGGAATTTGT
RPL37A	GTGGTTCCTGCATGAAGACAGTG	TTCTGATGGCGGACTTTACCG

Supplementary information Table 3 Cardiac progenitor marker antibodies

Marker	Source
NKx2.5	Abcam #ab91196
Islet1	Abcam #ab109517
Gata4	Abcam #ab134057
PDGFRα	Abcam #ab203491
